# Comparing the Perspectives of Managers and Employees of Teaching Hospitals About Job Motivation

**DOI:** 10.5539/gjhs.v6n6p112

**Published:** 2014-07-15

**Authors:** Rafat Mohebbifar, Mohammad Zakaria Kiaei, Omid Khosravizadeh, Mohammad Mohseni

**Affiliations:** 1 Department of Health Management, School of Health, Qazvin University of Medical Sciences, Qazvin, Iran; 2 Hospital Management Research Center, Iran University of Medical Sciences, Tehran, Iran; 3 Health Management and Economics Research Center, Iran University of Medical Sciences, Tehran, Iran

**Keywords:** job motivators, employee, managers, teaching hospitals

## Abstract

Recognition of career motivators and understanding of managers and employees in prioritizing them, in order to plan incentives for this understanding, can play an important role in increasing productivity and creating harmony between the goals of the organization and staff. This study was done to survey the importance of career motivating factors from perspective of employees and managers in educational hospitals of Iran. In this study 269 from a total of 1843 employees of educational hospitals in Qazvin province of Iran were selected through Quota-Random sampling and studied along with all 49 Managers. Lawrence Lindale questionnaire with 10 factors where used in order to determine motivational priorities. The results indicated that among the 10 studied motivational factors, from employees’ viewpoint; “Good wages”, “Good Working Conditions” and “Job Security” have the greatest roles in motivating employees. In the context of perspective agreement amongst employees and managers, the results showed 20 percent agreement. In this study, results of “Independent T” test showed a significant difference in comparison, between prioritizing employees’ view and prediction of managers in the factors of “Job Security” (p = 0.031) and “Interesting Work” (p = 0.001). With respect to increase disagreement in the views of managers and employees as compared to previous studies, Managers need to pay more attention to cognition of motivational factors and make their viewpoints closer to actual motivational need of their employees. Attention to this fact can be a great help to the growth and productivity of the organization, making the organizational and individual goals closer and also keeping managers safe from execution of constant and undue motivational patterns.

## 1. Introduction

Being the crucial core of health system, human resources are subject to indicate the main causes of poor quality in health services and adversely improving the expected health care (Hongoro & McPake, 2004; McQuide, Kolehmainen-Aitken, & Forster, 2013) In this regard, human resources for health are clearly a prerequisite for health care (Anand & Barnighausen, 2004). Today in Human Resources Management (HRM), one of the interesting topics is supplying employees’ needs in regard to motivation. Motivation Regarding the management principles incentive package is a mixed criteria to fulfill the staffs’ demands which is an appropriate induction to produce effective and efficient results, create a positive working environment for successful execution of predicted programs and to improve the quality of healthcare services (Bessell, Dicks, Wysocki, & Kepner, 2009; Lambrou, Kontodimopoulos, & Niakas, 2010; Misfeldt et al., 2013). This is while lack of explicit and clear policies for human resource management and paying not adequate attention to occupational motivational factors can threaten the capacity of health care systems in reaching their goals (Dussault & Dubois, 2003). Concerning the fact that commitment and motivation have a high positive correlation (Battistelli, Galletta, Portoghese, & Vandenberghe, 2013; Gaki, Kontodimopoulos, & Niakas, 2013) and also the distinguishing features and services Health and Treatment Center workforce offer, this issue is undoubtedly of a great importance. It can also have deep effects on the quality of services

Motivation is an inner force and a state which encourages individuals for a certain activity. The important thing for an individual or a group as a motive may not be important to another person or group (Gardulf et al., 2005). The basic condition for individuals participation and cooperation with an organization is career motivation and job satisfaction, which is subsequently resulted in increasing productivity, individual commitment to organization and self-motivation (De Cooman, Stynen, Van den Broeck, Sels, & De Witte, 2013; Mrugank & Ashwin, 2005).

The mutual understanding between managers and employees and the agreement on the importance and priority of different kinds of job motivators, provides the basis of harmonizing the organizational goals and employees demands which is the most important step in the organizations effectiveness (Amirtash, 1999).This statement is constant with conducted surveys on employees and managers in American industries in different decade such as 1949s and the results demonstrated that there is significant disparity between employees and managers’ point of view. Lawrence Lyndal’s primary research in the late 1940s, 1980s and 1990s also showed similar results indicating that what employees are looking for were significantly different from managers. Between the 10 motivational factors “Full appreciation for work done” was the most important factor for employees while this is the 8th factor in managers’ point of view. Also “Feeling “in” on things” was the 2nd most important factor whereas managers ranked that factor as 10th (Nelson, 2002). In addition to these vertical supports an employee should get from the workforce atmosphere, the correlation between coworkers is a key factor to reach the expected outcomes (Jungert, Koestner, Houlfort, & Schattke, 2013).

In a study entitled “Customer based employee motivation: understanding Job characteristics model” a significant correlation was observed between a raise in employees’ motive and the 3 dimensions of Job “Job’s Nature, Diversity of Skills, and Job’s Importance” (Mrugank & Ashwin, 2005). Sullivan in one of his studies showed that factors such as personal gratitude, promotions, public praise, and written gratitude are the most important factors which are effective in motivating employees (massodi asl, Behbahani, Nosratinejhad, & Gholamrezanejhad, 2010). Franco have evaluated effective motivating factors on health sector employees and have concluded that feeling pride, being effective, honesty in management and job security are among job motivation factors (Franco, Bennett, Kanfer, & Stubblebine, 2004).

Moody and Pesut in their research express that application and expansion of motivation theory in the nursing profession needs a special leadership and a strategic management to support a job motivation culture in health care organizations (Moody & Pesut, 2006). In another study, job security, effective leadership, effectively addressing employees’ demands, processes of evaluating the ideal performance, and good relations have been known as motivational factors influencing the performance of hospital staffs. Also we can attribute some of the factors involved in enhancing staffs’ motivations to appropriate management and organization in the hospital (Mbindyo, Gilson, Blaauw, & English, 2009).

Despite the vast amount of research done on motivation, researches on the most important job motivation factors in educational hospitals (especially comparison between views of employees and managers) are very limited.

Even though hospitals as a service organization with its own structure and complexity are key pillars of the healthcare and treatment system and indeed its main tool are human resources. Therefore recognition and analysis of motivational factors of employees of these organizations according to its crucial role in providing public health is of utmost importance. This study has been done in order to compare the importance of job motivators from the perspective of the managers and employees of hospitals in one of medical science universities of Iran, located in province of Qazvin, which has educational and healthcare hospitals. At the same time, this study wants to compare the results of two time period study 2002 and 2012, from the same population. With reflection and appropriate feedback lead the studied managers to efficiency and effective management of their affiliated hospitals.

## 2. Method

This study is a descriptive - analytic study which is done sectional in 2012 on all of employees and managers of an educational and healthcare hospital affiliated to a medical science university of Iran located in province of Qazvin. From a total of 1843 employees, 269 employees have been chosen through a form of Quota-Random sampling. Thus initially each hospital was categorized and then sample volume was divided proportional to the employees of that hospital and also in every Hospital, employees were grouped in 3 categories: Physicians, Technical Staff, and Support-Service Staff.

Physicians: including General Practitioners, Specialists, Pharmacists and Dentists.
Technical Staff: including Nurses, Midwives, Paramedics, Health workers, Assistant Health workers, Operating Room Personnel, Laboratory staff, and Radiology staff.
Support-Service Staff: including Administrative and Financial affairs Staff, and Service contractors’ employees.

A Managerial census was conducted and all 49 managers from different hospitals and departments such as, the Hospital President, Hospital Manager, Educational Deputy (Deputy director of education/vice president of education), Heads of Departments, Matrons, Support Managers (Administrative, Financial, and Service Affairs and officials in various departments of hospitals), were studied.

Information gathering tool was Lawrence Lindahl questionnaire (1949) used in 2002 study. This questionnaire contains 10 motivational factors, which has been designed separately for managers and employees. To determine reliability of the questionnaires the test-retest Method have been used and solidarity in employees’ motivational priorities questionnaires was equal to r = 0.84 and Managers’ was equal to r = 0.89 (Raeissi & Mohebbi far, 2006).

To prioritize studied factors, rank 1 was intended for the most important and 10 intended for the least important factor; the rest 2 to 9 are intended without repeating choice. Any factor whose mean was closer to 1 was of more importance. Based on this model employees were asked to rank their own motivational factors based on the most to least important from 1 to 10 and their managers were also asked to answer the questionnaire based on their view of their own employees perception and rank the 10 motivational factors based on same assumption as above. Data were analyzed using SPSS software and statistical tests “One way ANOVA”, and “independent T test”.

## 3. Results

After analyzing the data, the following results were obtained.

Calculating the mean of the Ranks given from employees’ viewpoint, “Good Wages”, “Good Working Conditions”, and “Job Security” had the first three ranks and “Feeling “in” on Things” become last. Based on managers’ conjecture and prediction, “Good Wages”, “Job Security”, and “Good Working Conditions” had the first three ranks and “Interesting Work” become last.

Furthermore, the results of the “Independent T test” for the factor “Job Security” (p = 0.031 and df = 316 and t = 216) and the factor “Interesting Work” (p = 0.001 and df = 316 and t = 47.3) showed a significant difference between the employees’ opinion and managers’ conjecture and prediction.

In terms of studying the agreement between the viewpoints of employees and managers in the context of job motivators, the results indicate that the agreement rate was 20% (Table 3).

**Table 1:** Demographic information of managers and employees

Demographic variable		Managers		employees	

		Number	Percent	Number	Percent
**sex**	Woman	29	63%	184	69.4%
	Man	17	37%	81	30.6%
**Marital Status**	Married	41	87.2%	210	79.2%
	Single	6	12.8%	53	20.8%

**Table 2:** Work experience of employees and management experience of managers

		Number	Percent
**Work Experience of employees**	1-10 years	128	50.6%
	11-20years	91	36%
	21-30years	34	13.4%
	Less than 5 year	15	38.5%
**Management experience of managers**	5-14years	15	76.9%
	15-20years	9	23.1%

**Table 3:** Ranking of Job Motivators based on the views of studied employees and managers in 2012

Job motivators	Employees		Managers	

	Mean±SD	Ranking	Mean±SD	Ranking
Good wages	4.15±2.98	1	3.80±3.04	1
Good working conditions	4.85±2.85	2	5.08±2.78	3
Job Security	4.97±2.89	3	4.02±2.51	2
Personal loyalty to workers	5.59±2.70	4	5.08±3.10	4
Tactful discipline	5.61±2.75	5	5.91±2.03	7
Full appreciation for work done	5.63±2.66	6	5.53±2.50	5
Promotion and growth opportunities	5.96±2.71	7	6.02±2.71	8
Interesting work	5.96±3.10	8	7.61±2.80	10
Sympathetic understanding of personal problems	6.19±2.64	9	5.59±2.46	6
Feeling “in” on things	6.29±2.70	10	6.57±2.55	9

On the other hand the ranking of the job motivators on the basis of the categories in which the employees were grouped indicated that from Physicians’ viewpoint, “Good Wages”, “Good Working Conditions”, and “Promotion and Growth Conditions” were the three most important factors and “Feeling “in” on things” was the least important one. From Technical Staff’s viewpoint, “Good Wages”, “Good Working Conditions”, and “Job Security” were the three most important factors and “Interesting Work” was the least important one. From Support-Service Staff’s viewpoint, “Good Wages”, “Job Security”, and “Interesting Work” were the three most important factors and “Feeling “in” on things” was the least important one (Table 4).

**Table 4:** Ranking of Job Motivators from studied employees’ viewpoint based on the groups they’re categorized to

Job motivators	Physicians		Technical Staff		Support-Service Staff	

	Mean±SD	Ranking	Mean±SD	Ranking	Mean±SD	Ranking
Good wages	3.32±2.48	1	3.9±2.82	1	4.76±3.21	1
Good working conditions	4.56±2.80	2	4.65±2.88	2	5.31±2.8	4
Promotion and growth opportunities	4.72±2.82	3	5.95±2.57	7	5.44±2.83	6
Job Security	4.92±2.87	4	4.85±3.01	3	5.03±2.7	2
Full appreciation for work done	5.52±3.04	5	5.79±2.71	6	5.39±2.44	5
Interesting work	5.64±3.58	6	6.43±3.05	10	5.20±2.95	3
Tactful discipline	5.76±2.74	7	5.44±2.63	4	5.84±2.95	8
Personal loyalty to workers	6.56±2	8	5.46±2.59	5	5.67±3.01	7
Sympathetic understanding of personal problems	7.08±2.23	9	6.19±2.67	9	5.96±2.67	9
Feeling “in” on things	7.16±2.11	10	6.09±2.66	8	6.37±2.80	10

Also the results of “One way ANOVA” test, showed that there is a significant relationship between the rating of the factors “Good Wages” (p = 0.031 and F = 3.52) and “Interesting Work” (p = 0.01 and F = 4.67) among the three working groups (physicians, technical staff and support-service staff).

In the present study, ANOVA shows that in the conjecture and prediction of managers and prioritization of job motivators there is a significant relation between the factor “Good Working Conditions” and management experience of studied managers (p = 0.045 and F = 97.2). Based on this test, there is a significant relationship between work history of managers and their conjecture and prediction of rankings of employees’ job motivators in the factors “Feeling “in” on things” and “Full appreciation for work done”.

Also results of the “T test” indicates that in context of employees’ marital status and their ranking of motivational factors there is a relation between the factor “Promotion and Growth Opportunities” and their marital status (p = 0.048, df = 261, and t = 1.98).

## 4. Discussion

Intrinsic and extrinsic incentives are crucial factors in job satisfaction but first matter to be concerned is identifying the context of this complex topic and its related factors (Ringelhan, Wollersheim, Welpe, Fiedler, & Spörrle, 2013).The research findings indicate that between the 10 studied motivational factors; based on staff’s point of view and factors prioritization are “Good Wages”, “Good Work Conditions” and “Job Security” have the greatest role in motivating employees (Gaki, Kontodimopoulos, & Niakas, 2013). This result is consistent with the results of a similar study on the same population in 2002 (Raeissi & Mohebbifar, 2006). whereas in another study factors “Job Security” and “Good Working Conditions” had the most importance for employees and the factor “Good Wages” had the sixth place (Amirtash, 1999). Yet in most studies conducted in different time periods, economic factor have been more important than other factors(Nelson, 2002), it is worth mentioning that work motivation might exhibit a nonlinear pattern over time and the effective factors are obviously subjected to change (Navarro, Curioso, Gomes, Arrieta, & Cortes, 2013).

However, on the conjecture and prediction basis of managers for ranking the motivational factors of their employees, the results of this study showed factors “Good Wages”, “Job Security” and “Good Working Conditions” for work are the most important factors which are similar to the results of the year 2002. But in terms of conjecture and prediction of managers about the least important factors, the results of this study showed that “Interesting Work”, “Feeling “in” on things” and “Promotions and Growth Opportunities” is the least important factors respectively. Whereas the results of the 2002 study shows that “Interesting Work”, “Sympathetic Understanding of Personal Problems”, and “Feeling “in” on things” were the least important factors (Raeissi & Mohebbifar, 2006).

In the extent of agreement between employees and managers about studied motivational priorities, this study shows that extent of this agreement was 20 percent which is identical to Amirtash’s study in Iran (Amirtash, 1999). The study conducted in educational hospitals of Qazvin University of Medical Science in the year 2002 the extent of agreement between managers and employees about studied motivational factors was 40 percent (Raeissi & Mohebbi far, 2006). Also in a study conducted by Lyndal the extent of agreement between managers and employees in the industries of America about the motivational factors has been almost zero and there was a significant disagreement between studied managers and employees about ranking of the questioned motivational factors (Franco et al., 2004). It’s interesting that all of the factors that employers considered them as the least important job motivators for workers were of the most important motivational factors for workers (Nelson, 2002).

Comparison on the agreement between managers and employees in the various categories indicates that there is a harmony about the studied motivational priorities to the extent of 30 percent between managers and physicians, 20 percent between managers and technical staff, and 30 percent between managers and support-service staff.

But the noteworthy fact is that in the 2002 study, in the same studied population, a significant disagreement is observed about the most important motivational factor in the “Physicians” category. So that now the factor “Good Wages” is the most important motivational factor and “Feeling “in” on things” is the least important. Whereas in the year 2002 “Interesting Work” and “Good Wages” were respectively the most important and the least important factor for the physicians. It’s important to mention that in the present study, the factor “Good Wages” had the 1st priority (most important factor) in all three categories that staff were grouped in, which indicates the importance of economic factors from the perspective of studied categories in the present time period. However, job motivational factors can vary at different times due to economic, political, and social circumstances and employees’ demands.

In a study conducted on the context of effective factors on motivating employees of public hospitals and private hospitals in Iran, the results indicated that spiritual factors are of more importance in the public hospitals compared to private hospitals (Abzari, shaemi, Pourmiri, & Azarbaijani, 2011) and human resources issues are the most important factor in promoting or impeding productivity (Nayeri, Nazari, Salsali, & Ahmadi, 2005). Studies show that materialistic and non-materialistic factors affect staffs’ motivation. In this study, it has been found that managers’ appreciation, job security, training, adequate salary, and acceptable conditions are among motivational factors in staffs of Health and Treatment Center (Dieleman, Cuong, Anh, & Martineau, 2003). What is Important is that due to increase in the extent of agreement between managers and employees compared to the previous period of time, managers need to pay more attention on understanding the job motivational factors and make their views closer to their employees’ real motivational needs. Different motivating plans have been designed to achieve the highest organizational outcomes (Parker, 2013; Taylor, 2013) because attracting and retaining the elite and efficient human resources is a challenge today’s managers are involved in. Therefore paying attention to this matter can be of great help to the growth and productivity of organization and making organizational and individual goals closer and save managers from implementation of constant and undue motivational patterns.
